# Oral Health-Related Quality of Life in a Paediatric Population in the Dominican Republic

**DOI:** 10.3390/jcm13092449

**Published:** 2024-04-23

**Authors:** David Ribas-Perez, Carlos Muñoz-Viveros, Angel Luis Formoso-Veloso, Francisco Jesus Carrillo-Sanchez, Luis El Khoury-Moreno, Julio Torrejon-Martinez, Antonio Castaño-Seiquer

**Affiliations:** 1 Department of Stomatology, University of Seville, 41004 Seville, Spainpacocarrillo11@gmail.com (F.J.C.-S.); lelkhoury@us.es (L.E.K.-M.); jtorrejon1@us.es (J.T.-M.); acastano@us.es (A.C.-S.); 2 Kerr Corporation, Orange, CA 91766, USA; cmunozviveros@yahoo.com

**Keywords:** oral health-related quality of life (OHRQoL), Child Oral Health Impact Profile-19 Short Form (COHIP-19SF), Dominican Republic

## Abstract

**Introduction:** During the summer of 2019 and within the framework of a social dentistry program carried out in the low-income town of San Francisco de Macorís (Dominican Republic), a descriptive study was carried out on oral health-related quality of life (OHRQoL), aiming to find out the oral health status of a population of children in the aforementioned Dominican city. **Objective:** The aim of this study was to describe the oral health status of a child population and its relationship with the quality of life perceived by these children in the aforementioned population of San Francisco de Macorís in order to develop an specific oral health program taking into account not only the existing oral health status but also the perceptions and feelings of the child population in this regard. **Method:** A descriptive cross-sectional study was carried out on a representative sample of children who were examined on their oral health status, following WHO guidelines, by professionals from the University of Seville (Spain) together with professionals from private practice (USA) and students from the Universidad Católica Nordestana (UCNE, Dominican Republic). Likewise, the children’s parents voluntarily completed the Oral Quality of Life questionnaire COHIP-19 in its culturally adapted Spanish version. **Results:** For this purpose, 94 children with a mean age of 10.34 (SD 3.38) were observed in our study following WHO recommendations for oral health studies and evaluating OHQoL using the specific questionnaire validated in Spanish COHIP-19 in its short format (SF). The results show a state of oral health with a significant prevalence of caries (80.9%) and a DMFT of 1.70 (SD 1.90). The OHQoL perceived by these children shows that pain, bad breath or feeling sad because of the condition of their teeth were the factors with the worst evaluation score. **Conclusions:** The conclusion that mainly emerges from this study is that caries continues to be the main problem to be solved (more than other variables studied, such as malocclusion or fluorosis), and this ailment also causes pain, dysfunction, and bad breath and is therefore perceived as a problem to be solved in the children of this Dominican city.

## 1. Introduction

Oral health is an indivisible part of the general health of any child. As we know, depending on the age of the child, they can be in a stage of primary, mixed, or permanent dentition. According to the Global Burden of Disease Study, in 2017, more than 530 million children worldwide had dental caries in their primary teeth. Traditionally, dental treatment of primary dentition has not been given sufficient attention, even though it can cause a large number of infections with their significant associated repercussions. There is also a clear relationship between caries in the primary dentition and its appearance in the permanent dentition [1,2].

Caries can have a significant impact on children, families, and societies. The disease begins in the primary teeth, may continue in the permanent teeth, and has an inescapable impact on overall health and oral quality of life throughout life. Caries may be related to other common childhood diseases, mainly due to risk factors in common with other non-communicable diseases, e.g., high sugar intake, and with diseases related to other health disorders such as obesity [3,4].

Dental problems may make chewing and sleeping difficult and restrict children’s life activity. Severe dental caries is associated with growth deficiencies, and in addition, caries can become an economic burden on the family and society; treatment of caries for extensive dental repair is particularly costly and unaffordable for many families [5].

All this can be obviously related to an inadequate oral quality of life, since the functions performed within the oral cavity can be altered and, in many cases, severely hindered or even prevented by oral disorders such as caries, which are easily preventable. On the other hand, the self-esteem of children suffering from oral diseases can be affected by this alteration of oral functions [6,7].

When establishing a situational analysis prior to the implementation of specific oral health programs, it is necessary to carry out epidemiological surveys to evaluate the human and economic resources necessary to carry out the program, thus better linking the program to the real and perceived needs of the population [8].

Traditionally, cross-sectional oral health studies have been carried out with a description of the health–disease status of the community, with data showing caries, periodontal diseases, and/or malocclusions to be the dominant conditions [9]. Recent studies have shown that other sociocultural factors, such as self-esteem and self-perception in community health, should also be considered for the structuring, design, and implementation of this type of oral health program. This is where the inclusion of oral quality of life surveys comes in for the prior and much-needed situational analysis.

A variety of questionnaires have been developed to measure oral quality of life for adults [10,11,12]. However, because children and adolescents have different quality of life issues compared to adults [13], several instruments have been developed in recent decades to measure oral quality of life in paediatric populations, despite the difficulties associated with the development and validation of such instruments [14].

These include the Oral Health Outcome Scale for 5-year-olds, the Paediatric Oral Health-Related Quality of Life Measure [15,16], and the Child Oral Impacts on Daily Performance Index [17]. However, the most frequently used self-completed quality of life scales for children are the Children’s Perceptions Questionnaire (CPQ) and the Children’s Oral Health Impact Profile (COHIP) [18,19].

The COHIP (Community Oral Health Improvement Plan), which was originally developed to assess “oral–facial well-being” across a range of ages (8–15 years) and ethnicities [19,20,21,22], is also a comprehensive and well-validated questionnaire for determining children’s OHRQoL. It contains 34 questions and 5 subscales (oral health, functional well-being, socio-emotional well-being, school environment, and self-image). The COHIP questions cover areas in the area of oral and maxillofacial health, as well as the inclusion of positive aspects of oral quality of life such as self-confidence and looking attractive).

The COHIP Short Form (COHIP-SF) 19 is an abbreviated version of the scale, developed in 2012, which contains 19 items and 4 subscales (self-image, oral health, functional well-being and socio-emotional well-being). In this abbreviated form, the psychometric properties of the original version are well maintained, and it can be administered more quickly, which facilitates the assessment of oral quality of life in clinical studies. For the interpretation of this questionnaire, we have, on the one hand, to analyze the final score of the questionnaire. On the other hand, we must also analyze the average score of each dimension and the score of each question individually [19,20,21,22,23,24].

In certain areas of the world that are more socioeconomically maligned, poorer oral health has been shown, and we assume that this results in poorer quality of life. Studies such as the one we have carried out in children to demonstrate this relationship between poor oral health and a low quality of oral life, which in principle would be a presupposition, are lacking.

The objective of the present study was to describe the oral health status of a population of children in the Dominican Republic (in terms of caries, fluorosis, and malocclusions) and its relationship with the oral quality of life perceived by these children using the COHIP 19 SF, with the final objective of carrying out an oral health program focused on the population studied.

## 2. Materials and Methods

### 2.1. Study Type and Settings

A cross-sectional study was carried out in the city of San Francisco de Macorís (Dominican Republic), Urbanización Vista del Valle, the subjects of which were 94 children between 4 and 16 years of age. These children attended the Dental Outreach Program co-organized by professionals from private practice (USA), the University of Seville, and the Universidad Católica Nordestana (UCNE, Dominican Republic) and were selected during the summer of 2019. All the children whose parents agreed to participate in the study and who did not present any type of severe systemic pathology that could alter the study were considered.

For the start of the measurements, written informed consent was requested from the parents or legal guardians, considering the international provisions of the Declaration of Helsinki (modification of Edinburgh 2000).

### 2.2. Data Collection

Data collection was carried out in two stages: first, clinical examinations were performed in portable dental units to obtain diagnoses of oral health status, applying the methodology recommended by the WHO in its book *Oral Health Surveys Basic Methods*, fourth edition [25]. Following these indications, caries, fluorosis (Dean’s index) and malocclusion indices were obtained.

In a second phase, the children’s parents completed the COHIP-19SF questionnaire that assessed levels of perception of Quality of Life related to oral health. Specifically, this questionnaire assesses four dimensions (functional well-being, socio-emotional well-being, oral health and self-image).

This questionnaire was previously evaluated for face validity by two examiners in a pilot test to assess its comprehension and to compare the different scores obtained with the theory. It contains 19 questions with a single response option on a 5-point Likert scale (ranging from never to always) and includes information on associated sociodemographic factors (age, sex, birth place).

### 2.3. Study Variables and Statistical Analysis

To conduct the statistical analysis for the oral health status and levels of perception of oral health-related quality of life, the means, standard deviation, frequency distribution, and percentages were calculated.

Relationships between variables were evaluated using the Chi-square test to test for statistical significance, assuming a statistically significant association when the p value was less than 0.05. All estimator values were adjusted from the sample design. The statistical program SPSS version 27 for Windows was used for the analysis.

## 3. Results

Of the 94 children seen in the operation, the mean age was 10.34 (SD 3.38). To facilitate the analysis, we grouped the children into small (under 6 years), medium (between 6 and 12 years), and large (over 12 years, up to 16 years). Of these three groups, the most frequent was the medium group with more than 50% of the children seen (57.4%) (Table 1).

A similar number of boys and girls (55.3% of children) were seen, almost all of whom were from San Francisco de Macoris (74.5%) and to a lesser extent from adjacent neighbourhoods.

Regarding dental anomalies, fluorosis was very rare (only clearly perceptible in 5.4% of the children) and it can be affirmed that more than half of the children had orthodontic needs (55.4% with slight or moderate malocclusions), as seen in Table 2.

The prevalence of caries found was significant (80.9%) with a mean DMFT of 1.70 (SD 1.90) and a dft of 1.86 (SD 2.04) for the total sample, although it is true that this index needs to be analyzed by age (Table 3).

When analysing each of the questions in the COHIP SF-19 questionnaire, we see the distribution in percentage of each answer to each question, as well as the mean and SD. In each of the 19 questions that make up the COHIP19 SF questionnaire, there are five possible answers that can be given, using a Likert scale from 0 to 4 where 0 corresponds to never and 4 to always (in terms of the frequency of occurrence with respect to the question asked). For each question, a mean was found with its corresponding standard deviation, as shown in Table 4.

For the analysis of this table and taking into account the results obtained from the measure of centralization that can provide us with the most information (mean with its SD), we see that there is a generalized tendency for the answers to be never or almost never, in many cases comprising almost 80% of the answers to this question (this is true for questions p1 (77.6%), p2 (77.6%), p7 (81.9%), p9 (78.7%), p11 (80.8%), p12 (83%), p13 (84%), and p14 (84%).

More focused responses with more frequent values of 1 and 2 were given for the questions between p16 and p19, with values ranging from 75.6% for p16 to 61.7% for p19.

Negative values with answers of 3 and 4 were almost not seen in the sample, the most striking being for question p6 with a value of 5.3% for the answer of always.

**Figure 1:**
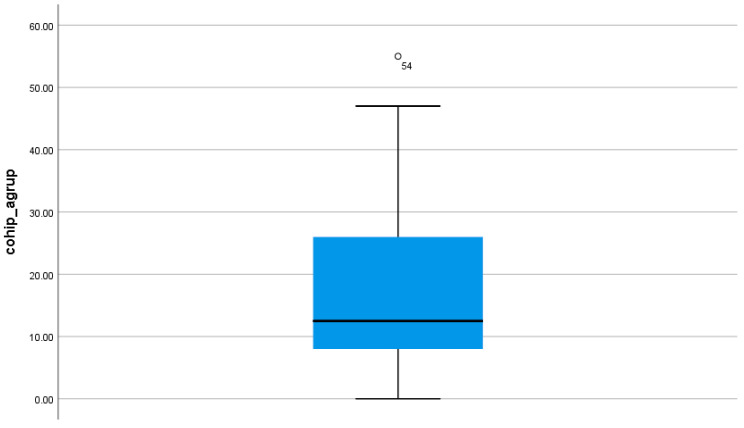
Final COHIP-19 cluster plot for the sample.

The overall mean COHIP-19 SF for all the patients was 14.01, with an SD of 16.73. This gives us a mean COHIP-19 value with an acceptable self-perception of their oral health, as can be seen in Figure 1. In this same graph, it can be seen that the median is even lower than that (12.10), meaning that more than 50% of the respondents show very good values of self-perception of their oral health.

Within the areas of study that can be encompassed according to the COHIP 19 questions are oral health (from question p1 to p5), functional well-being (p6, p7 p9, p10 p11, p12 p14 and p19), socio-emotional well-being (p13, p16, p17 and p18) and self-image (p8 and p15). If we analyze the mean of each of these subareas, we can see that the mean of oral health is 0.97 (SD 1.044), that of functional well-being is 0.84 (SD 0.88), that of socio-emotional well-being 0.92 (SD 1.086), and that of self-image 0.93 (SD 0.78).

When we relate each of the answers to each question with the different sociodemographic variables and pathologies studied, we can assess whether the relationship between them is statistically significant or not (it will be when the *p* value is <0.05 (Table 5). For example, with respect to age, there is statistical significance in questions p4, p5, p6, p11, and p18. Regarding sex, the *p* value was less than 0.05 in questions p1, p4, p7, and p8. The population of origin was not a variable that caused statistically significant differences to appear with respect to the questions of the COHIP-19 questionnaire.

**Figure 2:**
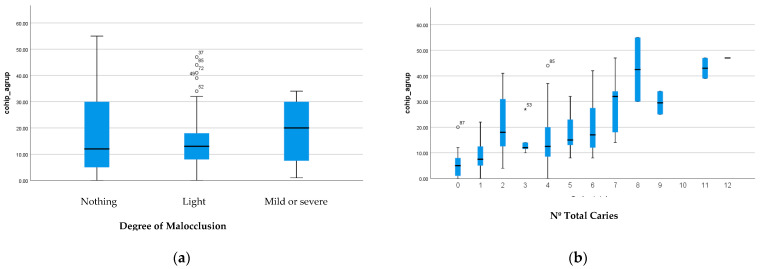
Relationship between degree of malocclusion and (**a**) number of total caries and (**b**) final COHIP score14.

With regard to caries and its relationship with the questions, there are statistically significant differences in questions p1 to p8, p10, and p15. In this case, moreover, the higher the number of caries, the worse the overall COHIP-19 scores, as can be seen graphically in Figure 2a.

With regard to malocclusion, there are only differences in question p9. It can also be seen that the existing difference in terms of caries is not seen in terms of malocclusion, with the means being similar in the children’s responses regarding their oral quality of life regardless of whether or not they have malocclusion (Figure 2b).

## 4. Discussion

One limitation of the study we have just presented is that it is a descriptive study in which the participants involved were those who attended the social project, so there was no sample selection. However, it does serve as a starting point for the future planning of oral health programs in the community.

### 4.1. Oral Quality of Life and Oral Pathology

In our study, the prevalence of caries was 80.9%. Taking (as a reference) the age of 12 years for comparison between the different studies, the prevalence is somewhat lower, with a percentage of 68.6%. In neighbouring countries, the prevalence values are very different, with data ranging from 35% in the study of Antigua and Barbuda to similar data in studies such as one in Haiti in 2005 [26]. If we compare them with data from the European Union, the prevalence values are far from those in countries such as Sweden, with 5.4%, or Spain, with 11.6% [25,26,27] (Table 6).

Access to healthcare should also be considered. Studies carried out in Sweden, the USA, Canada, and Australia [28,29,30,31] show that it is possible to achieve a significant reduction in the prevalence of dental caries when preventive strategies and improved accessibility to healthcare are taken into account.

As in our study, there are different studies that present similar results in terms of the correlation between the state of oral health and the socio-economic level of the individuals and in the demand for dental treatment. Although dentists are the main agents responsible for oral health, it is argued that there is also a duty of society to promote oral health education strategies, especially when there is an impact on quality of life and general health [32,33,34].

In this context, we believe that oral health education initiatives for young children implemented in general paediatrics and in the school setting should be valued. On the other hand, we also consider the education of the general population on the impact of oral disease on the quality of life when aesthetic and functional characteristics are compromised to be of great relevance.

Once again, we argue that prevention of childhood caries is essential, not only as a clinical problem for oral health professionals but also as a necessity in terms of oral health policy. Like other types of healthcare, considering the needs of children and families in oral healthcare will produce greater satisfaction and better compliance with medical recommendations [35,36,37,38].

As has been advocated in medical education and other health professionals’ education, in the practice of dentistry, attitudes may be trained and developed according to patient-centred clinical models, which advocate consideration of the patient and family’s needs, respect for their preferences and lifestyles, and participation in decision making for appropriate treatment plans. Patient-centred clinical methods and the attitudes consonant with them on the part of healthcare professionals result in an approach suitable to the child and his or her family as well as sound reasoning about aspects of quality of life [39,40,41,42,43,44,45,46,47].

The availability and accessibility of oral healthcare therefore depends not only on parental information and knowledge but also on the ability and financial resources of the family. In another study conducted in Italy and published in 2013 [48], it is stated that dental treatments are mainly provided by private health professionals; therefore, oral healthcare is mainly financed by direct payments from families or to a lesser extent through public schemes or private health insurance.

It is concluded that this fact endangers the most disadvantaged socio-economic populations because they do not manage to take care of their health; consequently, it leads to decreased resistance to oral and other diseases. Additionally, in Canada, there are discrepancies in access to oral healthcare because it is not included in the Canadian National Health Service, so the lower socio-economic classes have worse caries rates [49,50,51]. Thus, oral and dental health may be integrated into overall health promotion programs for families, using principles similar to the common risk factor approach [52,53,54,55].

Family-centred interventions aimed at health promotion suggest a suitable approach that can be incorporated into general health plans, always taking into consideration the specific characteristics of groups and communities [54,55]. Some studies argue that the family is the primary source of information on health, with mothers playing a fundamental role in modelling behaviours and attitudes related to healthy habits. In addition, the attitudes and values acquired in the early ages will influence the following stages and active responsibility for individual health [56].

Although health information in the family environment is developing, research also shows that some subjects never receive it. For this reason, the school has an important role in the creation of healthy environments and in the discussion of health-related topics [54,55].

As with caries, other oral pathologies also pose challenges regarding early interventions in prevention and health promotion. We also advocate for the need to resort to preventive strategies for malocclusion, since we recognize that some of the behaviours that are the basis of this type of pathology can be avoided and modified through the use of methodologies to provide information to parents and educators.

### 4.2. Oral Health Quality of Life: Early Intervention

Currently, there are no published studies regarding the Dominican Republic on oral health data in school children and no research on oral health-related quality of life. For this reason, it is difficult to compare the global results of the present study due to the lack of studies in the same geographic area. However, we know of different studies that assess oral health-related quality of life using scales such as the one used (COHIP-19 or ECOHIS) in early childhood in other countries—such as the USA, Turkey, Brazil, and China—either using non-probability samples or studying specific groups such as families of different socio-economic levels [57,58,59,60,61,62,63].

The results of our study confirm the hypothesis that alterations in oral health such as caries or alterations in occlusion affect the quality of life of preschool children and their families. Caries is the parameter that has the greatest impact on quality of life. As in other studies, malocclusions do not have such a great impact because there is no statistically significant association between COHIP 19 values and malocclusion [62,63,64,65,66,67].

This result shows that in the evaluation of oral health-related quality of life made by parents, they tend to consider caries first as a perceived health indicator. This fact is probably due to the consequences of caries perceived by parents as more severe and prolonged, causing them to neglect other oral health conditions that are as equally clinically complex [65].

The study by Martins-Júnior et al. conducted in Brazil with a population-based sample of preschool children corroborates the relationship between caries and oral health-related quality of life [59]. Analogous to what we found in our study, in this publication parents value caries in young children as a primary indicator of oral health-related quality of life. As in other age groups, oral health problems in children have an impact on quality of life because pain, discomfort, and functional limitation affect physical, psychological, and social capacity, which translates into difficulties in nutrition, language pronunciation, and socialization, as well as low self-esteem and irritability, among other issues [32,66].

The results in our work show an increase in the prevalence of caries with increasing age of the children, as it is related to the COHIP score. Thus, in older children, due to the permanence of the teeth for a longer period, they are subject to more aggressive pathologies and to developing more oral pathological symptoms. The age of the child influences the COHIP score, which agrees with the findings described in a Brazilian study [32].

The conclusion that older children have an increased likelihood of experiencing a negative impact on quality of life seems to be rooted in the fact that older children have more advanced-stage caries and have a greater and better ability to communicate with parents about the effect of oral health conditions on their quality of life.

This finding reinforces once again the need to consider early childhood oral health education as a priority. Caries lesions were associated with a negative impact on the quality of life of the students and their families, and traumatic dental lesions are also associated with worse quality of life [32].

Accordingly, the literature confirms that in children in whom a negative impact of oral health status on quality of life is found, the most frequently reported complaints are related to caries and to their developmental stage: pain, difficulty eating some foods and hot or cold drinks, sleep problems, irritability, and self-image problems when smiling [59,65,66,67].

Some authors argue that the mean COHIP score reflects an association between the presence of caries at different stages of development and impact on quality of life, with 40% to 69% of parents/caregivers reporting an impact on the child’s quality of life according to the demand (or lack thereof) for treatment [66].

These data show that the demand for dental treatment may depend on the perception of the child’s oral health conditions and eventual consequences [59]. In our study, perceptions of oral health were considered poorer than those of general health, which we can say is due to a concern on the part of parents who have been evaluated by oral health professionals because they are not as knowledgeable about the state of oral health because it is more specific. In addition, many of the children in our study had never been seen by a dentist, a fact that we verified empirically but which was also reported to us by those responsible for the school establishments.

For this reason, we believe that the commentary provided by us on the oral health of each child within his or her family will be very useful. Possibly, in this case, the socio-economic resources of the family will be reflected in the overall health of the child. The impact of oral disease on quality of life was perceived by parents to be negative; this occurred when the child presented caries or the need for treatment.

## 5. Conclusions

Although it must be taken into account that the data of this study correspond to a descriptive study without control of sample selection (since it was carried out on patients attending a social program), we can conclude that Dominican children have a prevalence of caries that is above the average of the surrounding countries and a perceived quality of oral health that does not correspond to this state of oral health.

These data show the need to carry out oral health programs focused on the treatment of caries (due to the damage it causes) rather than malocclusions, which do not have such a high impact on the quality of life of these disadvantaged populations.

## Figures and Tables

**Table 1:** Distribution of the sample by age group.

	Frequency (*n*)	Percentage (%)
Small < 6 years	15	16.0
Medium	54	57.4
Large > 12 years	25	26.6
Total	94	100.0

**Table 2:** Distribution of the sample by sex, origin, fluorosis, and malocclusion.

		Frequency (*n*)	Percentage (%)
Gender	Boys	52	55.3
	Girls	42	44.7
	Total	94	100.0
City	San Francisco de Macoris	70	74.5
	Santiago	16	17.0
	La Vega	8	8.5
	Total	94	100.0
Fluorosis (Dean index)	No fluorosis	82	87.2
	1 Dean	7	7.4
	2 Dean	4	4.3
	3 Dean	1	1.1
	Total	94	100.0
Malocclusion	None	42	44.7
	Light	48	51.0
	Mild or severe	4	4.3
	Total	94	100.0

**Table 3:** dft and DMFT in the sample.

	Mean	Dev.
dft (temporary)	1.86	2.040
DMFT (permanent)	1.70	1.905

**Table 4:** COHIP 19SF responses distribution; mean and SD.

Question	0 Never	1 Hardly Ever	2 Sometimes	3 Frequently	4 Always	Mean	SD
P1. Had pain in your teeth/toothache	44 (46.8%)	29 (30.8%)	16 (17%)	4 (4.3%)	1 (1.1%)	0.82	0.939
P2. Had crooked teeth or spaces between your teeth	52 (55.3%)	21 (22.3%)	15 (16%)	6 (6.4%)	0	0.73	0.952
P3. Had discoloured teeth or spots on your teeth	33 (35.1%)	31 (33%)	18 (19.1%)	7 (7.4%)	5 (5.4%)	1.15	1.145
P4. Had bad breath	29 (30.9%)	32 (34%)	20 (21.3%)	9 (9.6%)	4 (4.3%)	1.22	1.118
P5. Had bleeding gums	44 (46.8%)	22 (23.4%)	20 (21.3%)	6 (6.4%)	2 (2.1%)	0.94	1.066
P6. Been unhappy or sad	47 (50%)	20 (21.3%)	19 (20.2%)	3 (3.2%)	5 (5.3%)	0.93	1.148
P7. Missed school for any reason	31 (33%)	46 (48.9%)	13 (13.8%)	4 (4.3%)	0	0.89	0.796
P8. Been confident	32 (34.1%)	40 (42.5%)	19 (20.2%)	2 (2.1%)	1 (1.1%)	0.94	0.853
P9. Had difficulty eating foods you would like to eat	55 (58.5%)	19 (20.2%)	13 (13.8%)	7 (7.5%)	0	0.7	0.971
P10. Felt worried or anxious	37 (39.4%)	34 (36.2%)	17 (18.1%)	4 (4.3%)	2 (2%)	0.94	0.971
P11. Not wanted to speak/read out loud in class	60 (63.8%)	16 (17%)	17 (18.1%)	1 (1.1%)	0	0.56	0.824
P12. Avoided smiling or laughing with other children	64 (68.1%)	14 (14.9%)	14 (14.9%)	1 (1.1%)	1 (1%)	0.52	0.864
P13. Had trouble sleeping	64 (68.1%)	14 (15.9%)	15 (16%)	0	0	0.5	0.8
P14. Been teased, bullied, or called names by other children	66 (70.2%)	13 (13.8%)	11 (11.7%)	3 (3.2%)	1 (1.1%)	0.51	0.901
P15. Felt that you were attractive (good-looking)	26 (27.7%)	50 (53.1%)	17 (18.1%)	1 (1.1%)	0	0.93	0.707
P16. Felt that you look different	21 (22.3%)	47 (50.1%)	24 (25.5%)	2 (2.1%)	0	1.07	0.751
P17. Had difficulty saying certain words	29 (30.9%)	39 (41.5%)	20 (21.3%)	6 (6.4%)	0	1.03	0.885
P18. Had difficulty keeping your teeth clean	20 (21.3%)	45 (47.9%)	24 (25.5%)	5 (5.3%)	0	1.15	0.816
P19. Been worried about what other people think about your...	31 (33%)	32 (34%)	26 (27.7%)	5 (5.3%)	0	1.05	0.908
Social–emotional well-being subscale (p6–7, p10–12, p14, p16, p19)						0.92	1.086
Functional well-being subscale (p9, p13, p17–18)						0.84	0.88
Oral health subscale (p1–p5)						0.97	1.044
Self-image subscale (p8 y p15)						0.93	0.78

**Table 5:** Correlation between variables. * Statistical significance for d value at *p* < 0.05.

QUESTION	Age	Gender	City	Caries	Malocclusion
P1. Had pain in your teeth/toothache	0.263	0.026 *	0.144	0.012 *	0.115
P2. Had crooked teeth or spaces between your teeth	0.173	0.072	0.175	0.038 *	0.086
P3. Had discoloured teeth or spots on your teeth	0.158	0.101	0.186	0.000 *	0.489
P4. Had bad breath	0.030 *	0.034 *	0.593	0.001 *	0.160
P5. Had bleeding gums	0.001 *	0.080	0.328	0.042 *	0.454
P6. Been unhappy or sad	0.001 *	0.942	0.054	0.021 *	0.452
P7. Missed school for any reason	0.098	0.043 *	0.544	0.000 *	0.117
P8. Been confident	0.322	0.009 *	0.367	0.000 *	0.752
P9. Had difficulty eating foods you would like to eat	0.60	0.310	0.209	0.103	0.037 *
P10. Felt worried or anxious	0.155	0.661	0.450	0.046 *	0.106
P11. Not wanted to speak/read out loud in class	0.012 *	0.656	0.209	0.415	0.429
P12. Avoided smiling or laughing with other children	0.293	0.304	0.156	0.176	0.175
P13. Had trouble sleeping	0.330	0.463	0.322	0.179	0.330
P14. Been teased, bullied, or called names by other children	0.178	0.727	0.336	0.311	0.844
P15. Felt that you were attractive (good-looking)	0.246	0.382	0.137	0.026 *	0.567
P16. Felt that you look different	0.198	0.406	0.401	0.171	0.977
P17. Had difficulty saying certain words	0.298	0.824	0.911	0.152	0.986
P18. Had difficulty keeping your teeth clean	0.027 *	0.464	0.280	0.051	0.426
P19. Been worried about what other people think about your...	0.937	0.594	0.082	0.109	0.748
Socio-emotional well-being					
Functional well-being					
Oral health					
Self-image					

**Table 6:** Caries prevalence and DMFT in countries of the area at 12 years.

Country, Year	Caries Prevalence 12 Years	DMFT 12 Years
Bahamas 2000	54.5%	1.56
Antigua y Barbuda 2006	35.9%	0.90
Cuba 2000	54.5%	1.56
Dominican Republic 2008	--	8.66
Haiti 2005	71.9%	4.37
Jamaica 1995	41.0%	1.08
Puerto Rico 2011	69.0%	2.5
**San Fco Macoris 2019**	**68.6%**	**1.70**
Sweden 2020	5.4%	0.42
Spain 2020	11.6%	0.58

## Data Availability

Data will be available from the corresponding authors if required.
